# *In vitro* Antimicrobial Activity of Chlorquinaldol against Microorganisms Responsible for Skin and Soft Tissue Infections: Comparative Evaluation with Gentamicin and Fusidic Acid

**DOI:** 10.3389/fmicb.2017.01039

**Published:** 2017-06-08

**Authors:** Monica Bortolin, Alessandro Bidossi, Elena De Vecchi, Maura Avveniente, Lorenzo Drago

**Affiliations:** ^1^Laboratory of Clinical Chemistry and Microbiology, IRCCS Galeazzi Orthopaedic InstituteMilan, Italy; ^2^Laboratory of Clinical Microbiology, Department of Biomedical Sciences for Health, University of MilanMilan, Italy

**Keywords:** chlorquinaldol, gentamicin, fusidic acid, skin infections, time-kill curves, minimum inhibitory concentration, minimum bactericidal concentration

## Abstract

Skin and soft tissue infections *(SSTIs)* are a major therapeutic challenge for clinicians. The emergence of pathogens with decreased susceptibility to available therapies has become an emerging problem often associated with treatment failure. Hence, there is an urgent need for novel broad-spectrum antimicrobial agents. The purpose of this study was to assess the feasibility of chlorquinaldol as an alternative approach to currently used topical antibiotics for the treatment of skin and soft tissue infections. The activity of chlorquinaldol was investigated against a collection of bacterial isolates responsible for skin infections, including strains resistant to fusidic acid and gentamicin. After determination of MIC and MBC, time-kill experiments were carried out by counting colonies grown after 0, 3, 6, 9, 24, and 48 h of incubation with concentrations equal to ¼×, ½×, 1×, 2×, and 4× MIC of chlorquinaldol, gentamicin, or fusidic acid. Staphylococci resulted the Gram-positives most sensitive to chlorquinaldol, with MIC-values ranging from 0.016 to 0.5 mg/L. A lower activity was observed against Gram-negative bacteria, with 77% of the isolates being inhibited at concentrations ranging from 128 to 512 mg/L. Generally, in time-kill studies, chlorquinaldol showed a bactericidal activity at the higher concentrations (2×, 4× MIC) after 24–48 h of incubation. In conclusion, chlorquinaldol may represent a valuable alternative to conventional topical antibiotics for the treatment of skin and soft tissue infections.

## Introduction

Skin and soft tissue infections (SSTIs) are commonly occurring diseases with a wide-range of clinical manifestations varying from minor superficial to life-threatening infections (Ray et al., 2013). They may range from “simple uncomplicated” infections, such as erysipelas, cellulitis, simple abscesses, furuncles, and wound infections, to deeper “complicated” infections, such as necrotizing fasciitis, myositis, and gas gangrene.

The etiology of SSTIs is dominated by *Staphylococcus aureus*, but other common isolates include β-haemolytic streptococci, enterococci, Enterobacteriaceae, anaerobes, and *Pseudomonas aeruginosa* (Lipsky et al., 2007).

SSTIs are caused by microorganisms multiplication, leading to prolonged inflammatory response, delays in collagen synthesis and epithelialization, and tissue damage. Even though careful attention is paid to patients with acute infections, treatment often fails in chronic ones, where healing is hampered by the presence of bacterial biofilm (Bjarnsholt, 2013).

Despite high rate of morbidity and significant burden to the health care system, the management of skin lesions is still far from being completely effective and novel therapies are urgently needed (van Koppen and Hartmann, 2015).

Topical antibiotics are key components in the management of skin lesions. However, cases of sensitization are not uncommon (Bonamonte et al., 2014). Poor penetration and variable antimicrobial concentration can lead to resistance and scarce efficacy (Esposito et al., 2016). The emergence of pathogens with decreased susceptibility to available antibiotics has become an emerging clinical problem often associated with treatment failure, even with topical drugs of choice, such as fusidic acid (Heng et al., 2013). Fusidic acid monotherapy, in particular as topical preparation, is frequently used in dermatology and has been linked to the emergence of fusidic acid resistance in MSSA and MRSA (Heng et al., 2013).

Hence, there is an urgent need for novel broad-spectrum antimicrobial agents, which can be used to successfully treat cutaneous infections and overcome antibiotic resistance.

Chlorquinaldol, or hydroxydichloroquinaldine (5,7-dichloro-2-methyl-8-quinolinol), is a derivative of 8-hydroxyquinoline which is usually administered topically for the treatment of skin infections, alone or in combination with diflucortolone valerate (Maeder et al., 1983; Hoppe, 1988; Corrihons et al., 1991). In few past studies, chlorquinaldol has proven to be effective *in vitro* against a wide variety of fungi and a few bacterial species, and *in vivo* against a series of cutaneous conditions involving microbial infections or allergic skin conditions for which a supplementary anti-infective treatment, for prophylaxis or therapy, was indicated (Littman, 1955; Robinson and Hollander, 1956; Mann et al., 1960; Regös et al., 1979; Meyer-Rohn and Puschmann, 1980; Maeder et al., 1983; Kolev et al., 1987; Hoppe, 1988; Corrihons et al., 1991; Sikorski et al., 1992; Zlatkov et al., 1996). Despite these promising observations, no further studies about the antimicrobial activity of chlorquinaldol were performed and recent investigations are lacking.

The purpose of this study was to assess the feasibility of chlorquinaldol as a potential alternative approach to currently used topically applied antibiotics for the treatment of skin infections, including fusidic acid and gentamicin.

## Materials and methods

### Microorganisms

Clinically relevant strains isolated from SSTIs at the Laboratory of IRCCS Galeazzi Orthopedic Institute were used. In particular, methicillin-resistant *S. aureus* (MRSA, *n* = 9), methicillin-susceptible *S. aureus* (MSSA, *n* = 8), methicillin-resistant *Staphylococcus epidermidis* (MRSE, *n* = 13), *Streptococcus pyogenes* (*n* = 6), *Enterococcus faecalis* (*n* = 13), *Propionibacterium acnes* (*n* = 10), *Proteus mirabilis* (*n* = 11), *P. aeruginosa* (*n* = 12), *Escherichia coli* (*n* = 12), and *Enterobacter* spp. (*n* = 12) were used. Identification of the isolates was carried out on a Vitek2 Compact (BioMerieux, Marcy L'Etoile, France) and further confirmed by pyrosequencing (PSQ96RA, Diatech, Jesi, Italy).

### Drugs

Stock solutions of chlorquinaldol (MedChemtronica AB, Stockholm, Sweden), fusidic acid (Cayman Chemical, Ann Arbor, MI, USA), and gentamicin (MP Biomedicals, Santa Ana, CA, USA) were prepared in 95% ethanol (chlorquinaldol) or sterile water (fusidic acid and gentamicin) at concentrations of 5,000 mg/L and stored in aliquots at −20°C until use.

### Evaluation of minimum inhibitory concentration (MIC) and minimum bactericidal concentration (MBC)

The bacteriostatic and bactericidal properties of chlorquinaldol and its spectrum of activity were evaluated in comparison with gentamicin and fusidic acid.

The antimicrobial activity was assessed by determining the MIC and the MBC-values against the microbial strains described above. The MIC was determined by broth microdilution method, in accordance with EUCAST standard^1^. Briefly, a suspension in growth medium (Brain heart infusion broth, Biomérieux, Marci l'Etoile, France) was prepared for each bacterial strain with an optical density equal to 0.5 McFarland (1.5 × 10^8^ CFU/ml). For *P. acnes* broth was supplemented with 3% of defibrinated sheep blood (Liofilchem, Roseto degli Abruzzi, Italy). After obtaining a concentration of 10^5^ CFU/ml using appropriate dilutions, each suspension was inoculated in a 96-wells microtiter plate containing a serial 2-fold dilution of chlorquinaldol, fusidic acid, or gentamicin. MIC-values, corresponding to the lowest concentration exhibiting no visible bacterial growth, were read after 24 h of incubation at proper conditions (except for *P. acnes* that required 48 h). The MBC was determined by plating 10 μl from each well showing no turbidity onto agar plates. After incubation at proper conditions, MBC was read as the lowest concentration able to kill 99.9% of the initial inoculum.

### Time-kill curves

Killing curves were performed against one representative strains of each species described above. Briefly, a suspension in growth medium was prepared for each bacterial strain with an optical density equal to 0.5 McFarland (1.5 × 10^8^ CFU/mL). An aliquot was inoculated into test tubes containing 2 ml of growth medium alone (growth control) or containing each testing substance (see above) at different concentrations (¼×, ½×. 1×, 2×, and 4× MIC). Final concentration of bacteria was 5 × 10^5^–5 × 10^6^ CFU/mL. Tubes were incubated at 37°C in proper conditions. Microbial counts were performed after 0, 3, 6, 9, 24, and 48 h of incubation by plating 0.1 mL of a proper dilution of bacterial suspension onto agar plates. Results were expressed as Log_10_ (CFU/mL). The limit of count detection was 200 CFU/mL. Bactericidal activity was defined as a 3 Log_10_ decrease in CFU/mL (99.9% kill) of the initial inoculum. Bacteriostatic activity was defined as <99.9% kill.

Time-kill curves of gentamicin and fusidic acid were performed only on strains classified as susceptible according to EUCAST breakpoint^2^.

## Results

### Chlorquinaldol, fusidic acid, and gentamicin antibacterial activity

Tables 1, 2 show MIC and MBC ranges, while Tables 3, 4 show distributions of MIC and MBC-values for chlorquinaldol, fusidic acid, and gentamicin tested against Gram-positive and Gram-negative strains.

**Table 1:** MIC and MBC ranges for Gram-positive bacterial strains.

	**Chlorquinaldol**		**Gentamicin**		**Fusidic acid**	
	**MIC (mg/L)**	**MBC (mg/L)**	**MIC (mg/L)**	**MBC (mg/L)**	**MIC (mg/L)**	**MBC (mg/L)**
MRSA	0.125–0.25	0.125–4	2–>512	2–>512	0.016–0.032	0.125–2
MSSA	0.25–0.5	1–4	2–>512	2–>512	0.016–2	0.125–2
MRSE	0.016–0.5	0.032–4	0.25–512	0.25–128	<0.016–8	<0.016–64
*E. faecalis*	0.25–2	0.25–2	NT	NT	4–16	16–512
*S. pyogenes*	8–32	32–128	NT	NT	4–8	16–64
*P. acnes*	16–32	32–512	>512	>512	0.125–0.5	2–16

*NT, not tested because of intrinsic resistance to gentamicin*.

**Table 2:** MIC and MBC ranges for Gram-negative bacterial strains.

	**Chlorquinaldol**		**Gentamicin**	
	**MIC (mg/L)**	**MBC (mg/L)**	**MIC (mg/L)**	**MBC (mg/L)**
**GENTAMICIN-SUSCEPTIBLE STRAINS (MIC** ≤ **4 mg/L)**				
*E. coli*	8–128	>512	2–4	4–8
*Enterobacter* spp.	128–512	>512	2–4	2–8
*P. mirabilis*	512	>512	4	4
*P. aeruginosa*	128–512	512–>512	2–4	4–32
**GENTAMICIN-RESISTANT STRAINS (MIC** > **4 mg/L)**				
*E. coli*	8–512	>512	8–>512	8–>512
*Enterobacter* spp.	128–512	>512	64–>512	64–>512
*P. mirabilis*	32–512	256–>512	8–>512	16–>512
*P. aeruginosa*	32–256	128–>512	128–>512	256–>512

**Table 3:** MIC distributions (mg/L).

	**<0.016**	**0.016**	**0.032**	**0.064**	**0.125**	**0.25**	**0.5**	**1**	**2**	**4**	**8**	**16**	**32**	**64**	**128**	**256**	**512**	**>512**
**CHLORQUINALDOL**																		
MRSA					1	8												
MSSA						5	3											
MRSE		1	1	1	4	4	2											
*E. faecalis*						1	2	9	1									
*S. pyogenes*											3	2	1					
*P. acnes*												4	6					
*E. coli*											9				1	1	1	
*Enterobacter* spp.															3	6	3	
*P. mirabilis*													1			5	5	
*P. aeruginosa*													2		5	4	1	
**GENTAMICIN**																		
MRSA									5						1		1	2
MSSA									3	4								1
MRSE						4		1				1		3	3	1		
*P. acnes*																		10
*E. coli*									1	5	1	1				1	2	1
*Enterobacter spp*.									4	4				1			1	2
*P. mirabilis*										1	1			1	2	2	1	3
*P. aeruginosa*									1	2					1		2	6
**FUSIDIC ACID**																		
MRSA		5	4															
MSSA		1	6						1									
MRSE	3	1	2		1		1		2	2	1							
*E. faecalis*										1	6	6						
*S. pyogenes*										4	2							
*P. acnes*					4	5	1											

**Table 4:** MBC distributions (mg/L).

	**<0.016**	**0.016**	**0.032**	**0.064**	**0.125**	**0.25**	**0.5**	**1**	**2**	**4**	**8**	**16**	**32**	**64**	**128**	**256**	**512**	**>512**
**CHLORQUINALDOL**																		
MRSA					2	2	1		3	1								
MSSA								1	1	6								
MRSE			2		1	3	1	2	3	1								
*E. faecalis*						1	1	7	4									
*S. pyogenes*													3	2	1			
*P. acnes*													2	4	1	2	1	
*E. coli*																		12
*P. aeruginosa*															1	1	5	5
*Enterobacter* spp.																		12
*P. mirabilis*																1	3	7
**GENTAMICIN**																		
MRSA									1	3			1			1		3
MSSA									1	4	1		1					1
MRSE						3			1	1			1	1	3	2	1	
*P. acnes*																		10
*E. coli*										4	3	1				1	1	2
*P. aeruginosa*										1	1		1			1	2	6
*Enterobacter* spp.									4	3	1			1				3
*P. mirabilis*										1		1			1		5	3
**FUSIDIC ACID**																		
MRSA					2	1	1	2	3									
MSSA					2	1	1	1	3									
MRSE	1				2		3	2		1	1		2	1				
*E. faecalis*												1				5	7	
*S. pyogenes*												3	1	2				
*P. acnes*									2	6	1	1						

#### Gram-positive bacterial strains

Among Gram positives, staphylococci resulted the most sensitive cocci to chlorquinaldol, which was able to inhibit bacterial growth at concentrations ranging between 0.016 and 0.5 mg/L. MBC-values were highly variable: in 47% of cases they were equal or 2-fold the MIC, in 37% 4–8-folds the MIC, and in the remaining 16% they were 16–32-folds the MIC. Chlorquinaldol was effective at low concentrations against *E. faecalis*, presenting MIC and MBC-values ranging from 0.25 to 2 mg/L. MBC-values were always close to MIC-values (equal or 2-folds higher than the MIC). Modest inhibitory activity was observed against *S. pyogenes* and *P. acnes*.

All staphylococci with the exception of five MRSE strains resulted resistant to gentamicin. All *P. acnes* presented MIC and MBC-values higher than 512 mg/L. Staphylococci displayed the highest variability among the tested strains with MIC and MBC-values ranging from 0.25 to 1,024 mg/L.

All *S. aureus* strains were susceptible to fusidic acid, presenting MIC-values varying from 0.016 to 0.032 mg/L, with the exception of one MSSA isolate (MIC 2 mg/L). Eight strains of *S. epidermidis* were susceptible to fusidic acid, while five strains were found to be resistant. Propionibacteria were inhibited by low concentrations of fusidic acid, while higher MIC-values were observed for *E. faecalis* and *S. pyogenes*. Generally, MBC of fusidic acid were 4–128 times higher than the corresponding MIC.

#### Gram-negative bacterial strains

Gram-negative strains revealed a high heterogeneity in distribution of gentamicin resistance, so that susceptible and resistant strains are reported separately in Table 2. Strains with a MIC-value higher than 4 mg/L were 50% of *E. coli*, 75% of *P. aeruginosa*, 33% of *Enterobacter* spp., and 81% of *P. mirabilis*. MBC-values were close to MIC-values (equal or two times higher than the MIC), except for one *P. aeruginosa* strain (MIC 2 mg/L, MBC 32 mg/L).

Chlorquinaldol spectrum of activity revealed a lower activity against Gram-negative bacilli in respect to the Gram-positive tested bacteria, with 77% of the isolates being inhibited at concentrations ranging from 128 to 512 mg/L and 23% being inhibited at concentrations ≤32 mg/L. Of the latter group, 9 *E. coli* isolates showed a MIC-value of 8 mg/L, while 1 *P. mirabilis* and 2 *P. aeruginosa* isolates showed a MIC-value of 32 mg/L. MBC-values were ≥512 mg/L for 77% of tested strains (Table 4).

### Time-kill curves

**Figure 1:**
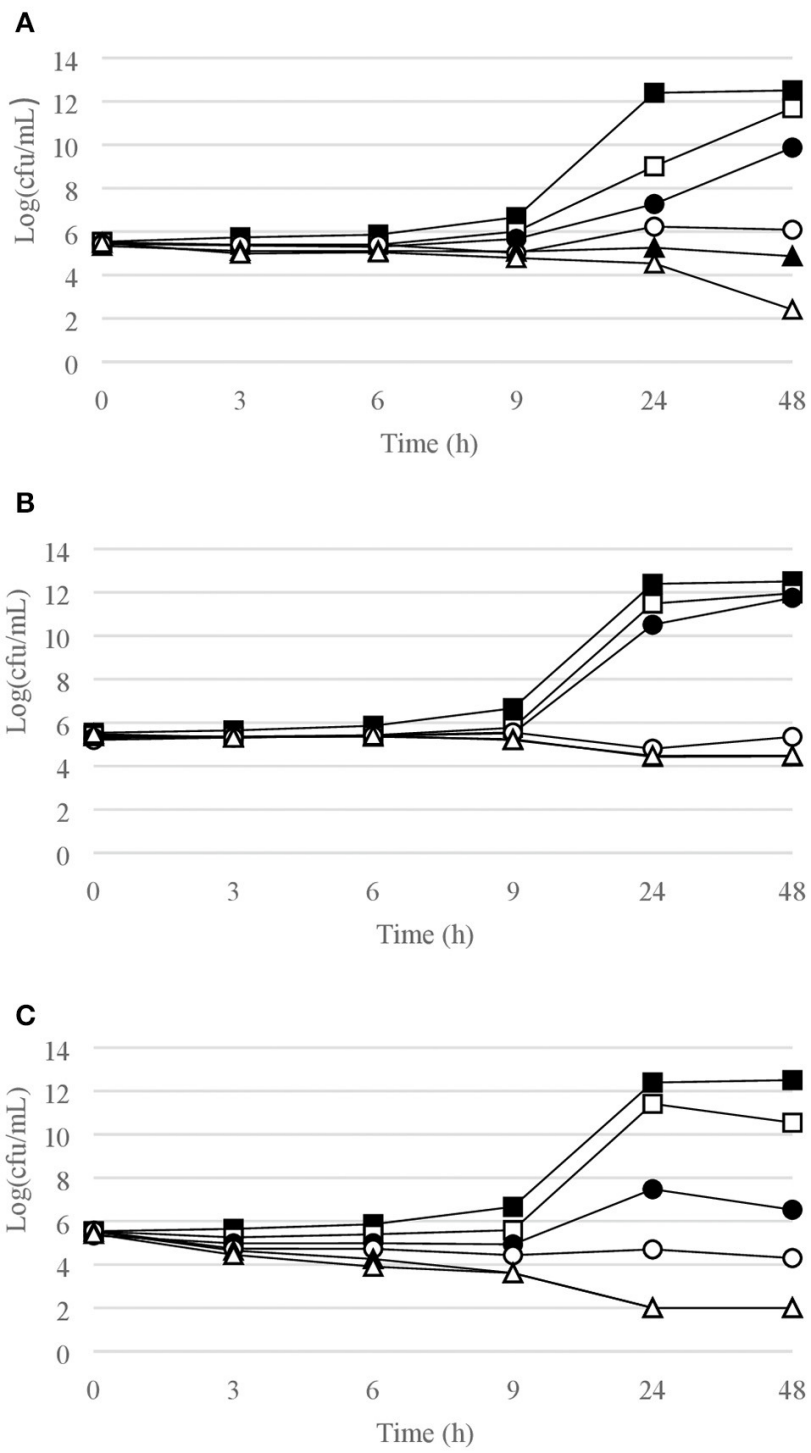
Time-kill curves for *S. epidermidis*. **(A)** chlorquinaldol; **(B)** fusidic acid; **(C)** gentamicin. Filled square, control; open square, ¼ × MIC; filled circle, ½ × MIC; open circle, 1 × MIC; filled triangle, 2 × MIC; open triangle, 4 × MIC.

**Figure 2:**
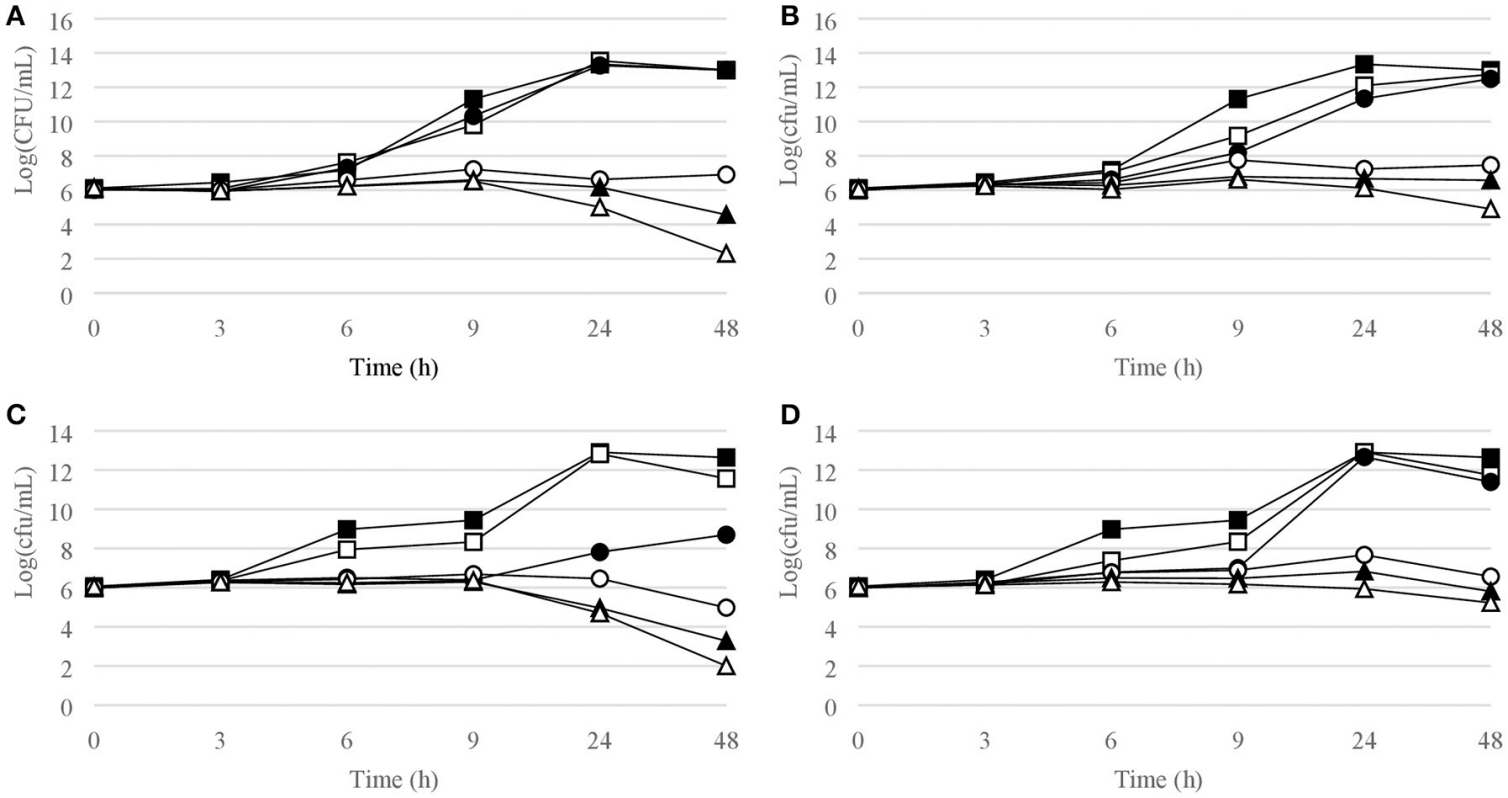
Time-kill curves for *S. aureus*. **(A)** chlorquinaldol, MSSA; **(B)** fusidic acid, MSSA; **(C)** chlorquinaldol, MRSA; **(D)** fusidic acid, MRSA. Filled square, control; open square, ¼ × MIC; filled circle, ½ × MIC; open circle, 1 × MIC; filled triangle, 2 × MIC; open triangle, 4 × MIC.

**Figure 3:**
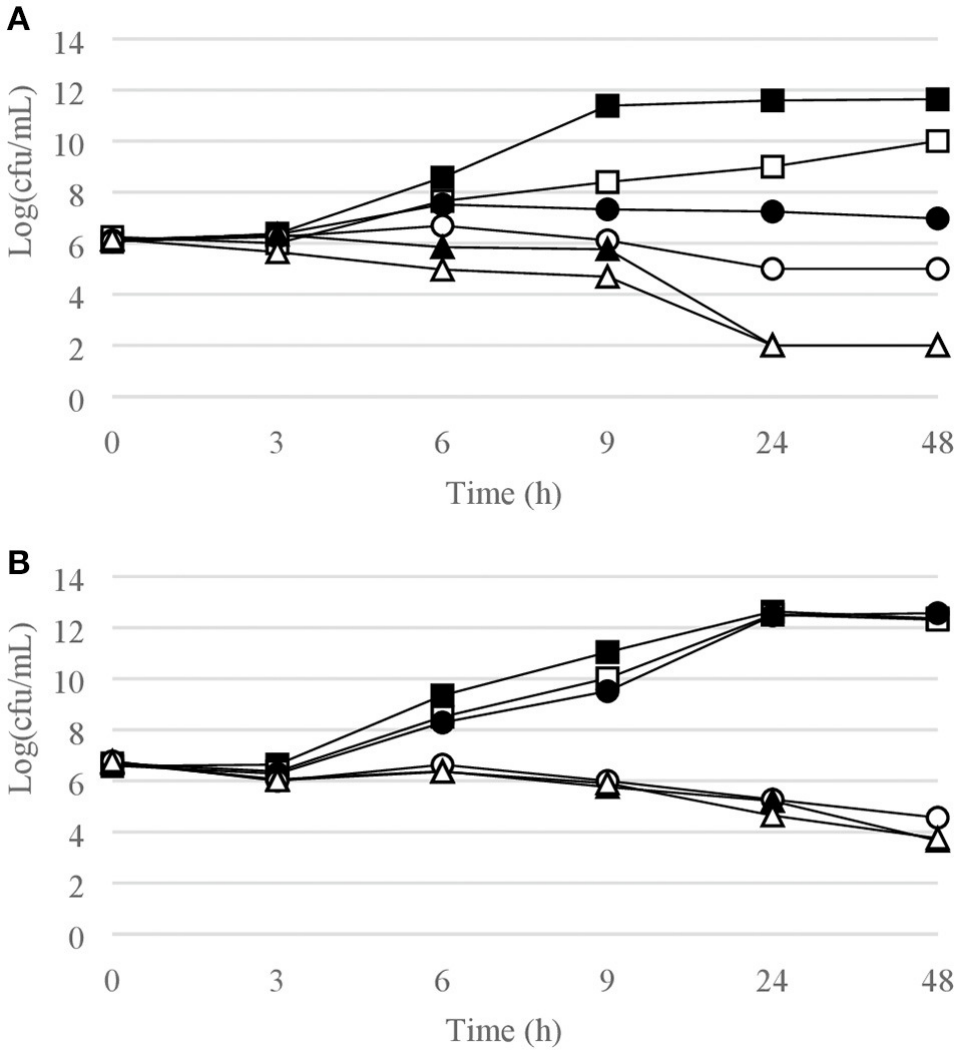
Time-kill curves for **(A)** *S. pyogenes*, chlorquinaldol; **(B)** *E. faecalis*, chlorquinaldol. Filled square, control; open square, ¼ × MIC; filled circle, ½ × MIC; open circle, 1 × MIC; filled triangle, 2 × MIC; open triangle, 4 × MIC.

**Figure 4:**
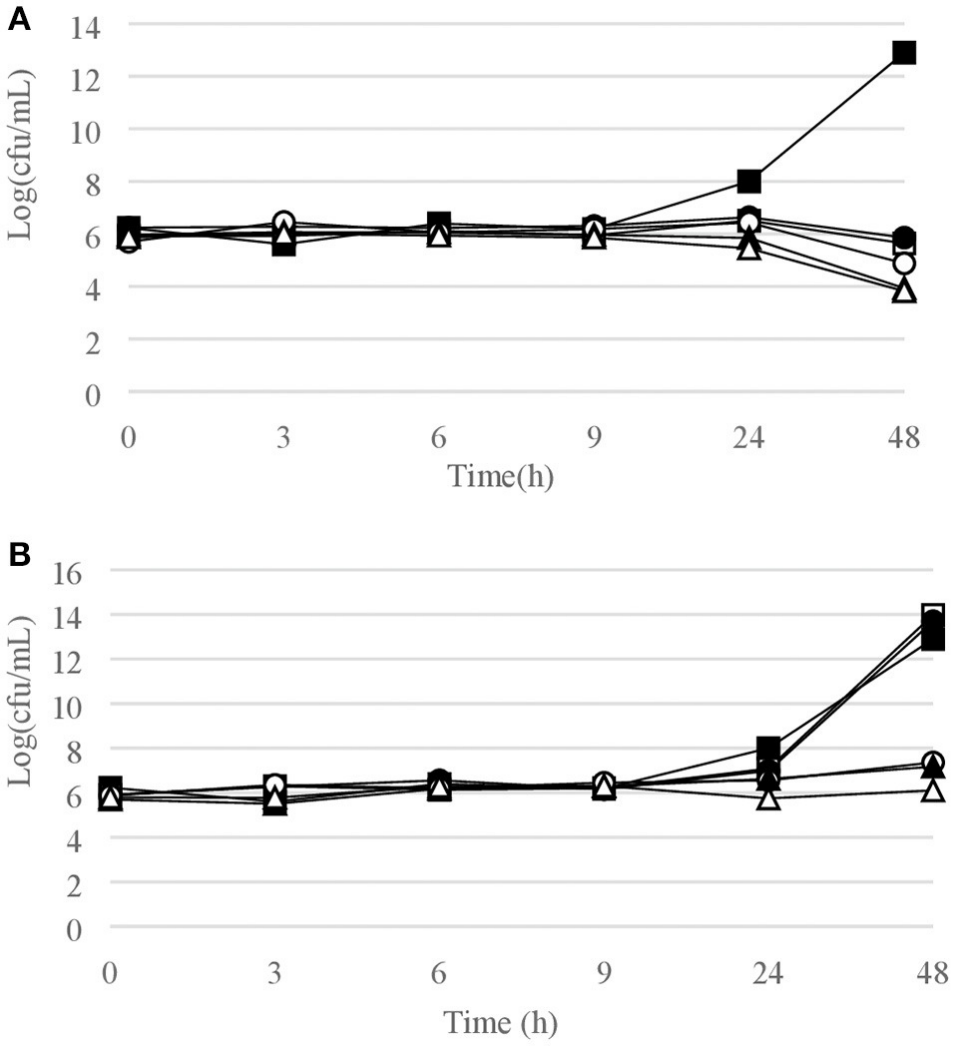
Time-kill curves for *P. acnes*. **(A)** chlorquinaldol; **(B)** fusidic acid. Filled square, control; open square, ¼ × MIC; filled circle, ½ × MIC; open circle, 1 × MIC; filled triangle, 2 × MIC; open triangle, 4 × MIC.

**Figure 5:**
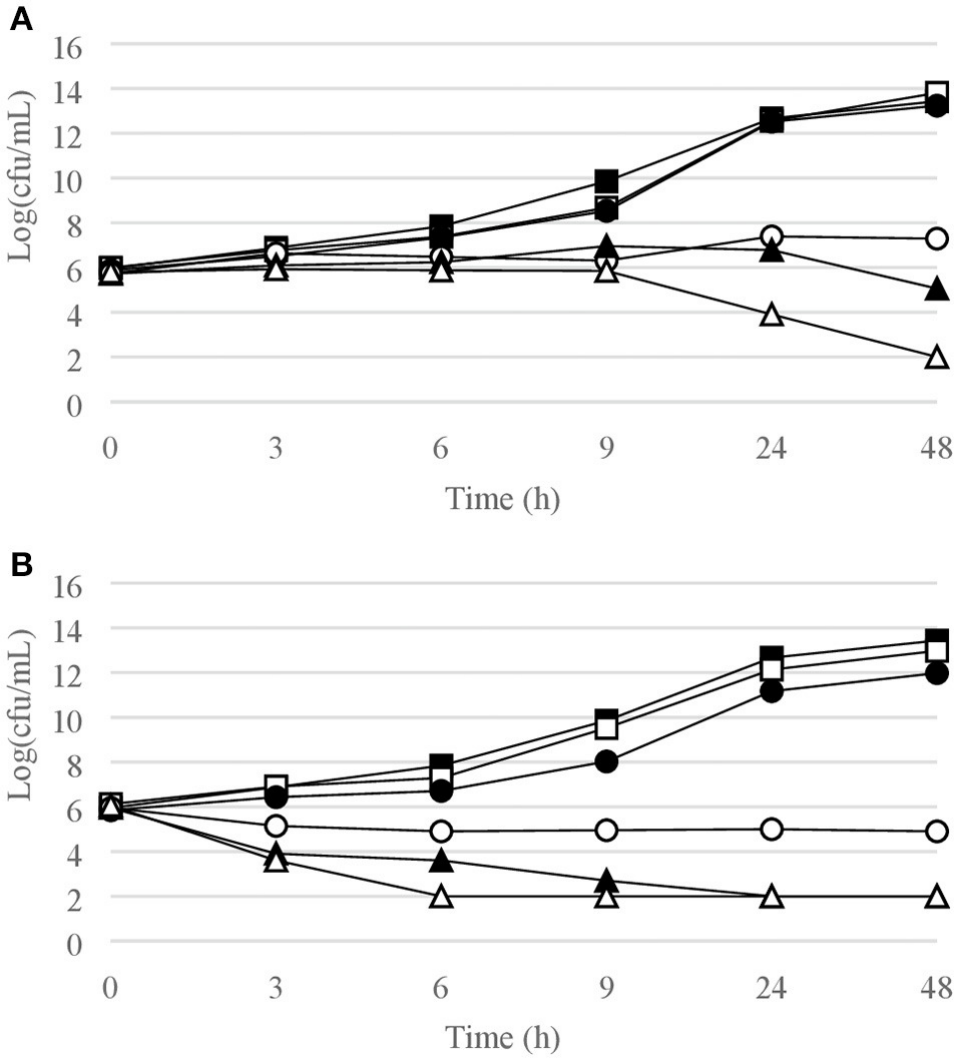
Time-kill curves for *P. aeruginosa*. **(A)** chlorquinaldol; **(B)** gentamicin. Filled square, control; open square, ¼ × MIC; filled circle, ½ × MIC; open circle, 1 × MIC; filled triangle, 2 × MIC; open triangle, 4 × MIC.

**Figure 6:**
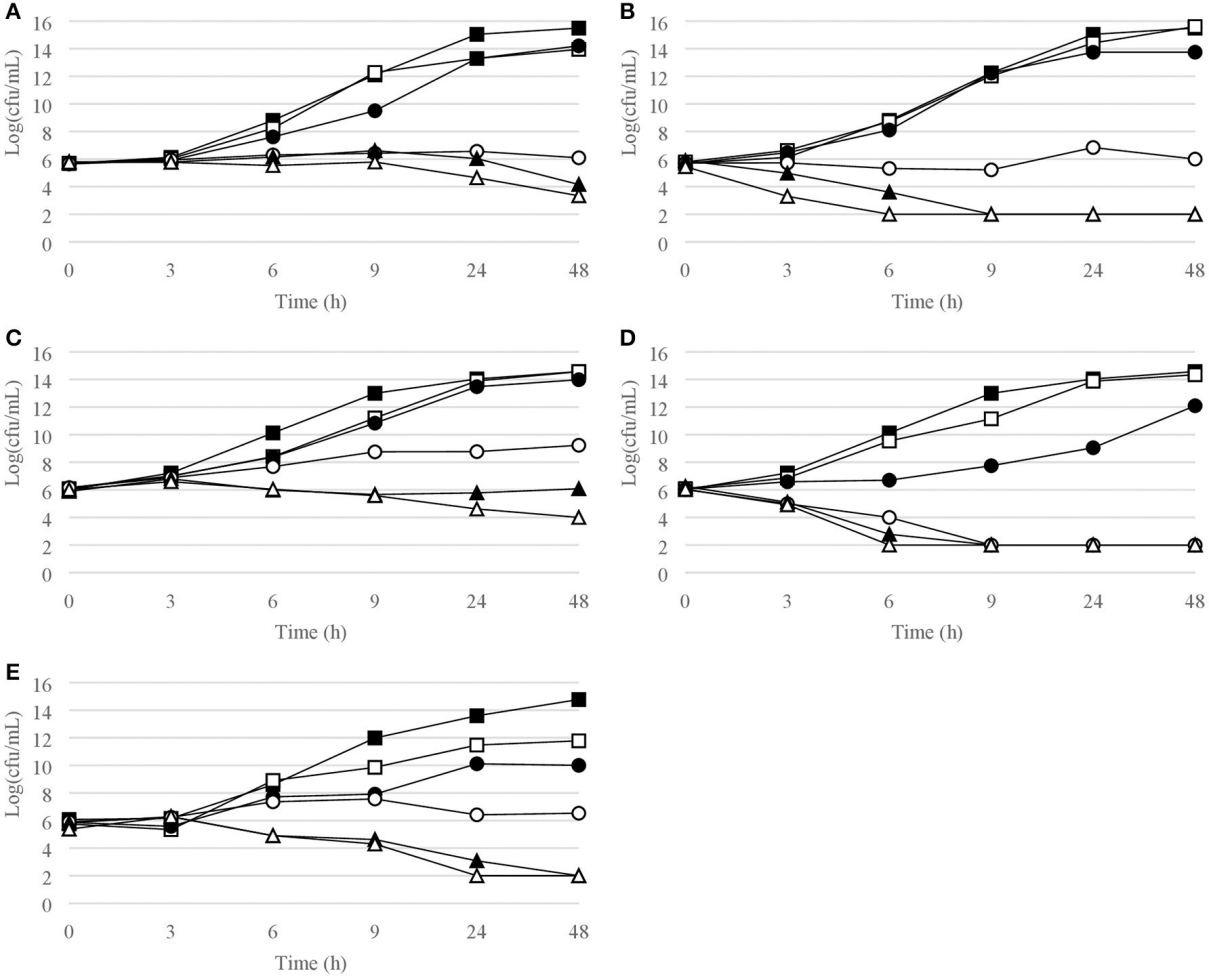
Time-kill curves for enterobacteriaceae. **(A)** *E. coli*, chlorquinaldol; **(B)** *E. coli*, gentamicin; **(C)** *E. cloacae*, chlorquinaldol; **(D)** *E. cloacae*, gentamicin; **(E)** *P. mirabilis*, chlorquinaldol. Filled square, control; open square, ¼ × MIC; filled circle, ½ × MIC; open circle, 1 × MIC; filled triangle, 2 × MIC; open triangle, 4 × MIC.

Figures 1–6 show the pattern of growth and kill by antibiotics of the various microbial species tested, at different concentrations of each of the tested antibiotics.

#### Gram-positive bacterial strains

Time-kill curves for Gram-positive strains are reported in Figures 1–4. Chlorquinaldol showed bactericidal activity, as defined by a 3 log_10_ reduction in viable count, after 48 h for *S. epidermidis* (4 × MIC), MSSA (4 × MIC), MRSA (4 × MIC), and *E. faecalis* (4 × and 2 × MIC), and after 24 h and 48 h for *S. pyogenes* (4 × and 2 × MIC). 2 log_10_ reductions were observed for MRSA (2 × MIC), *P. acnes* (4 × MIC), and *E. faecalis* (1 × MIC) after 48 h of exposure. Gentamicin (4 × and 2 × MIC concentrations) was bactericidal against *S. epidermidis* after 24 and 48 h. Fusidic acid was essentially bacteriostatic against staphylococci and *P. acnes*.

#### Gram-negative bacterial strains

Time-kill curves for Gram-negative strains are reported in Figures 5, 6. Chlorquinaldol displayed a bactericidal activity against *P. aeruginosa* after 48 h exposure (4 × MIC) and against *P. mirabilis* after 24 h (4 × MIC) and 48 h (2 × and 4 × MIC). 2 log_10_ reductions were observed for *E. coli* (4 × MIC) and *E. cloacae* (4 × MIC) after 48 h of exposure, and for *P. mirabilis* after 24 h (2 × MIC). Gentamicin was bactericidal against *E. coli* after 6 h (4 × MIC) and 9 h (2 × MIC), against *E. cloacae* after 6 h (2 × and 4 × MIC) and 9 h (1 × MIC), and against *P. aeruginosa* after 6 h (4 × MIC) and 9 h (2 × MIC).

## Discussion

SSTIs are common in both community and hospital settings. They present with a wide clinical spectrum, from superficial self-limiting infections to deep and potentially life-threatening ones (Ray et al., 2013). Gram-positive organisms, such as staphylococci and *S. pyogenes*, are the dominant organisms isolated in the acute infectious process, whereas Gram-negative organisms are more often seen in chronic or postoperative wounds (Cardona and Wilson, 2015).

In the recent years, there has been an increased incidence in SSTIs as a consequence of a number of factors, such as aging of the general population, higher incidence of immunocompromised patients and emergence of multidrug-resistant pathogens.

Topical antibiotics are key components in the management of SSTIs, but the emergence of pathogens with decreased susceptibility to available therapies has become an emerging clinical problem often associated with treatment failure. In this background, alternative treatments for patients infected with bacteria resistant to conventional antimicrobials are urgently required. Some possibilities comprise combination therapy or revival of older antimicrobial agents, for which resistance has not been developed yet.

One of these old drugs is chlorquinaldol, which is generally administered as an ointment in association with topical corticosteroids (usually 1% chlorquinaldol plus 0.1% diflucortolone valerate) for the treatment of SSTIs. Its percutaneous absorption is very small, narrowing the risk of systemic effects (Degen et al., 1979). Few past studies showed its efficacy *in vivo* against various skin and soft tissue diseases of infectious nature (Maeder et al., 1983; Hoppe, 1988; Corrihons et al., 1991). Nevertheless, recent investigations re-evaluating its antimicrobial activity are lacking and its mechanism of action is not clear yet.

Chlorquinaldol is a bihalogenated derivative of 8-hydroxyquinoline. Antimicrobial effects of 8-hydroxyquinoline and its derivatives, encompassing antibacterial, antimalarial, antiviral, antitubercular, and antiplaque activities have been previously reported (Prachayasittikul et al., 2013). It is assumed that they take advantage of their lipophilicity to penetrate bacterial cell membranes, where their antimicrobial action is probably related to chelating activities (Anjaneyulu et al., 1982; Hongmanee et al., 2007; Darby and Nathan, 2010). Metal ions play an important role in biological processes, and metal homeostasis is required for maintenance of cellular functions. Chelated metals become unavailable, inhibiting certain metabolic processes (Fraser and Creanor, 1975; Rohde et al., 1977; Chobot et al., 2011; Skrivanova et al., 2016). Until now, neither breakpoint nor pharmacodynamics data for chlorquinaldol, nor studies reporting MIC-values for a wide range of both Gram-positive and Gram-negative microorganisms are available. In this context, it is difficult to define a spectrum of activity for chlorquinaldol.

In the present study, chlorquinaldol exerted growth-inhibitory effects at different concentrations, depending on the species in exam. In particular, it seemed to be more effective against Gram-positive bacteria, especially staphylococci and enterococci, which are the most frequent microorganisms causing skin infections (Zlatkov et al., 1996; Lipsky et al., 2007).

Skrivanova et al. suggested that interspecies variability in the response to 8-hydroxyquinolines might be due to the different capacities of bacteria to accumulate metallic ions (Skrivanova et al., 2016). Hence, the role of metalloenzymes in different species should be explored to identify the mechanism of chlorquinaldol antibacterial action.

In this study, the most prominent effects exerted by chlorquinaldol were observed on staphylococci. In a past work, Mann et al. evaluated the antimicrobial activity of chlorquinaldol against 200 strains of *S. aureus*, reporting much higher MIC-values (6–100 mg/L) than those observed in the present manuscript (Mann et al., 1960). The reason for this discrepancy is unknown; we hypothesize that it may be due to less standardized methods respect to present international guidelines. In example, no informations about the inoculum are provided, nor about the manufacturer of chlorquinaldol, the exact formulation and the purity.

In the present work, inhibitory concentrations of chlorquinaldol were slightly higher than those of fusidic acid, but differently from this latter, activity of chlorquinaldol was mainly bactericidal. This activity was confirmed by the time-kill curves: chlorquinaldol was bactericidal for both *S. aureus* and *S. epidermidis*, killing more than 99.9% of the initial inoculum at 48 h at 4 × MIC concentrations, while fusidic acid displayed a classical bacteriostatic trend. Furthermore, five MRSE and one MSSA were resistant to fusidic acid while all MIC of the staphylococci tested for chlorquinaldol fell into a narrower range of action, always below a concentration of 0.5 mg/L. These data are of particular interest because of the long time documented emergence of fusidic acid resistance associated with topical monotherapy, which is five times higher than in oral treatment (Turnidge and Collignon, 1999; Howden and Grayson, 2006; Heng et al., 2013; Williamson et al., 2014). Though gentamicin showed a bactericidal effect at a concentration of 2 × MIC at 24 h against the tested staphylococci, only 20% of tested strains resulted susceptible to gentamicin. Indeed, high percentages of gentamicin-resistance in staphylococci isolated from skin infections have been reported by other authors, and nosocomial outbreaks of gentamicin-resistant *S. aureus* associated with the use of topical gentamicin were described in the past (Bint et al., 1977; Wyatt et al., 1977; Graham et al., 1980; Moet et al., 2007; Iwaki et al., 2011; Caraciolo et al., 2012). Moreover, use of gentamicin impregnated collagen sponges has been recently associated to an increased probability of sternal wound infection after cardiac surgery caused by gentamicin-resistant bacteria (Rapetto et al., 2016). As shown by time-kill curves, chlorquinaldol was bactericidal also against *E. faecalis* and *S. pyogenes*, at concentration of 2 × MIC, while no bactericidal effects were observed against *P. acnes*. All the tested *E. faecalis* strains were susceptible to chlorquinaldol at low concentrations, with an MBC-value equal or 2-fold the MIC-value. Considering the intrinsic resistance to gentamicin of this species and the poor activity of fusidic acid on our isolates, which is in line with literature review, chlorquinaldol can represent a valid choice to treat *E. faecalis* infections (Collignon and Turnidge, 1999).

Chlorquinaldol was effective against all the Gram-negative strains at high concentrations. We observed gentamicin resistance in more than 60% of the tested strains, which is higher than that observed in the SENTRY Antimicrobial Surveillance Program over a 7-year period (1998–2004) in SSTI (Moet et al., 2007).

Considering that, usually, topical formulations contain high concentrations of antibiotics, it may be hypothesized that chlorquinaldol levels at the infection site might be much higher than the MIC observed in this study.

In conclusion, chlorquinaldol, due to its wide spectrum of activity, would provide a valuable alternative for the treatment of SSTIs. Moreover, its use in combination with other antibiotics could be hypothesized in order to enhance antimicrobial activity and to limit the increasing resistance to topical antibiotics. Further, studies aiming to define a clinical breakpoint, evaluate synergy with other compounds and the ability to select for resistance after antibiotic exposure are advisable in the next future. Old studies pointed out chlorquinaldol efficacy against mycotic infections, so additional investigations to update its spectrum of activity against fungi would be also of great interest.

## Author contributions

LD conceived and designed the experiments. MB, AB, and MA performed the experiments. MB, AB, and ED analyzed the data. MB and AB prepared figures and graphs and wrote the manuscript. LD and ED revised the manuscript. All the authors read and approved the final manuscript.

### Conflict of interest statement

The authors declare that the research was conducted in the absence of any commercial or financial relationships that could be construed as a potential conflict of interest.

## Footnotes

^1^http://www.eucast.org/fileadmin/src/media/PDFs/EUCAST_files/MIC_testing/Edis5.1_broth_dilution.pdf

^2^http://www.eucast.org/clinical_breakpoints/
